# Supplementary material for: How Assad changed population growth in Sweden and Norway: Syrian refugees’ impact on Nordic national and municipal demography
Source: PLoS One. 2021 Jan 20;16(1):e0244670. doi: 10.1371/journal.pone.0244670 (PMC7816981; doi:10.1371/journal.pone.0244670)
Supplement: S1 Appendix — (DOCX) [file pone.0244670.s001.docx]

**Appendix 1: Comparisons with net migration trends from similar countries**Fig S1 compares our no-war scenarios with registered trends in net migration from neighbouring countries (indexed so 2010=100). In the Swedish case, the no-war scenario for Syria is most similar to that for Egypt, a country that also experienced the Arab spring but where this did not end in a civil war. For the majority of the other countries, the net migration trend did not increase quite as much as in our no-war scenario for Syria. This indicates that our results on the excess migration from Syria may be somewhat too low, and thus that the demographic effect of the Syrian war may have been larger in Sweden than our results indicate. In the Norwegian case, some of these flows are small and thus the yearly net migration fluctuations are large. However, our no-war scenario does not stand out compared to the net migration from neighbouring countries.


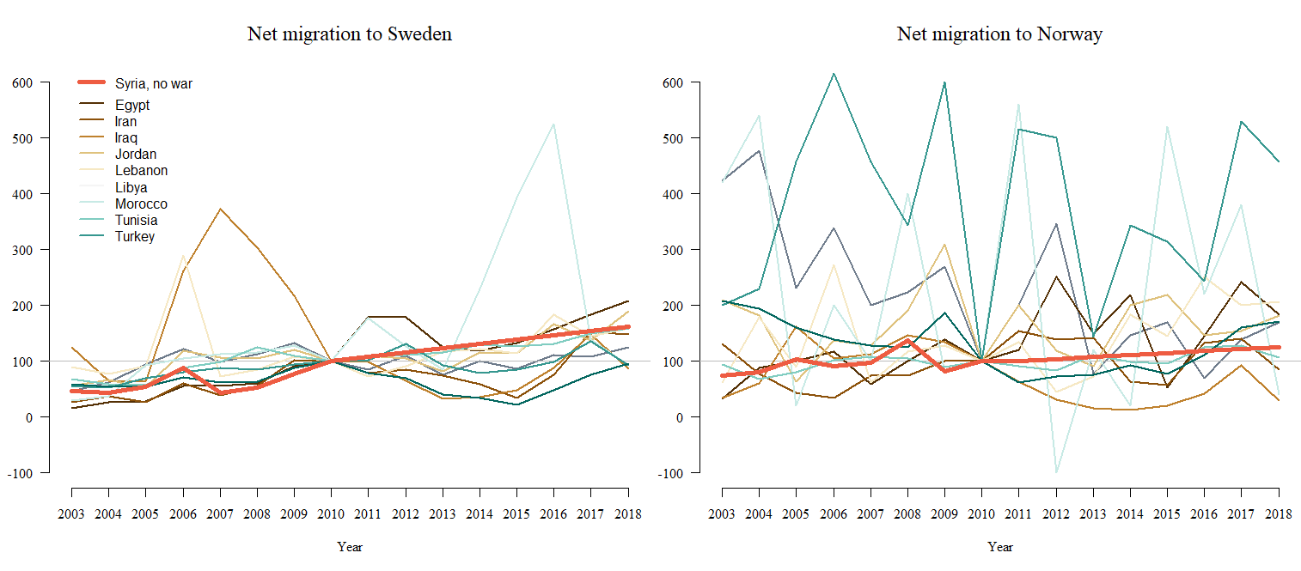


**Fig S1. Net migration from Middle Eastern countries to Sweden (upper panel) and Norway (lower panel), indexed (2010=100) and compared with no-war scenario for Syrian net migration (orange line).**
Sources: Statistics Sweden and Statistics Norway.
