# Supplementary material for: How Assad changed population growth in Sweden and Norway: Syrian refugees’ impact on Nordic national and municipal demography
Source: PLoS One. 2021 Jan 20;16(1):e0244670. doi: 10.1371/journal.pone.0244670 (PMC7816981; doi:10.1371/journal.pone.0244670)
Supplement: S2 Appendix — (DOCX) [file pone.0244670.s002.docx]

**Appendix 2: Net migration from other countries before and after 2015**

The large net migration of Syrians to Sweden and Norway in 2015/2016 may have had a ’crowding out’ effect on migrants of other nationalities. To investigate this, we have employed an extrapolation of the total number of migrants from other countries, based on data from before the Syrian refugee influx (i.e. 2003-2015). The results are shown in Fig. S2A. The red line shows net migration of Syrians, with the peak in 2016 (many of these arrived in the fall of 2015), and all other nationalities. The dotted line shows the average increase from 2003 to 2015, extended onwards to 2019.

The results differ markedly between Sweden and Norway. Whereas Sweden had higher net migration of non-Syrians after 2015 than the 2003-2015 trend suggest, the opposite is the case for Norway. Further, while the non-Syrian net migration has declined in Sweden after 2017, it has increased in Norway. These diverging trends may have several reasons. They may also be partly interrelated, since in this period many people moved from Norway to Sweden, affecting net migration in both countries.


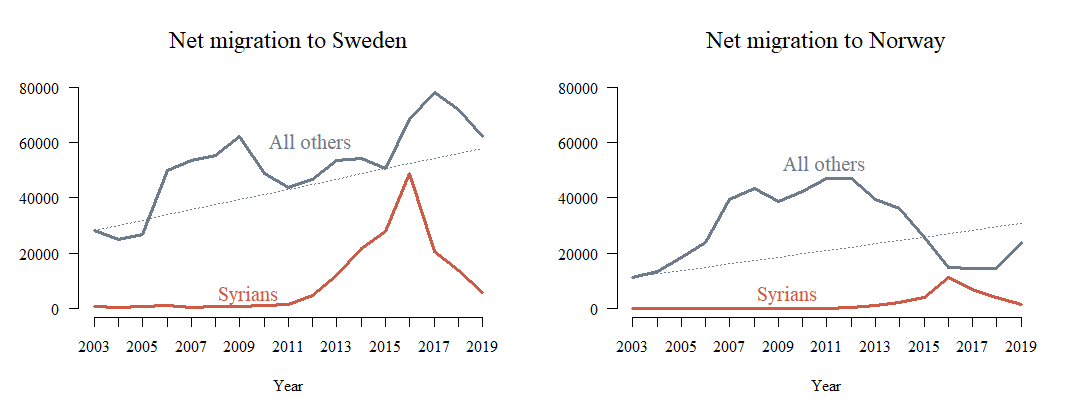


**Fig S2A: Net migration of Syrians and other citizens to Sweden (left) and Norway (right), 2003-2019**.

Sources: Statistics Sweden and Statistics Norway.

The effect of high Syrian net migration on other migration may go through several channels. One main channel is through employment: If the newly arrived Syrian occupy jobs that would otherwise be available for other immigrants who do not speak the Swedish/Norwegian language, the net migration of these others may decline. We may assume that this would primarily happen after a few years, since most refugees in Sweden and Norway follow introductory courses for refugees the first years after arrival, and that it primarily affects other (potential) migrants who do not speak a Nordic language.

A second important channel is through policy: Immigrant policies were tightened in most European countries in the aftermath of the 2015 asylum seeker flows, including in Sweden and Norway. We may expect that this tightening would have a relatively immediate effect on net migration, but only for Non-EU citizens since the free mobility within EU was not part of this policy tightening.

To investigate these possible mechanisms, we have divided the non-Syrian net migration into several groups: Nordic citizens (which we may assume were not very affected by the immigrant of Syrians – neither through the policy channel, nor through the employment channel since they do not compete for the same jobs), Non-Nordic EU citizens (which we may hypothesise were not affected through the policy channel, but might have been affected some years after 2015/16 through the employment channel), and net migration of non-Syrians from outside the EU (who may have been affected both through the policy and the employment channel).

Figure S2B shows how the net migration patterns for these three groups changed after 2015/16 in Sweden and Norway. The figure may indicate a crowding out effect for one group: In Sweden, Non-Nordic EU net migration did decrease in 2017, 2018 and 2019 – which may be an employment effect of an influx of Syrians into the Swedish labor market. On the other hand, a similar trend for this group is not found in Norway (rather, Non-Nordic EU net migration to Norway increased after 2017). Also for the net migration of Non-EU citizens, the trends in Norway are the opposite of those in Sweden: While the figures for Sweden indicate no effect of policy tightening the first years after 2015, the decrease after 2017 may indicate some effect through the employment channel. In Norway, the net migration of Non-EU citizens has been relatively stable since before the large influx of Syrian refugees in 2015.

In sum, it is hard to find clear evidence that the influx of Syrian refugees curbed migration from other countries. The patterns in Sweden and Norway are too divergent, even when dividing into subgroups who presumably are affected by different mechanisms, indicating that other factors than the influx of Syrians have played important roles for shaping these migration flows.


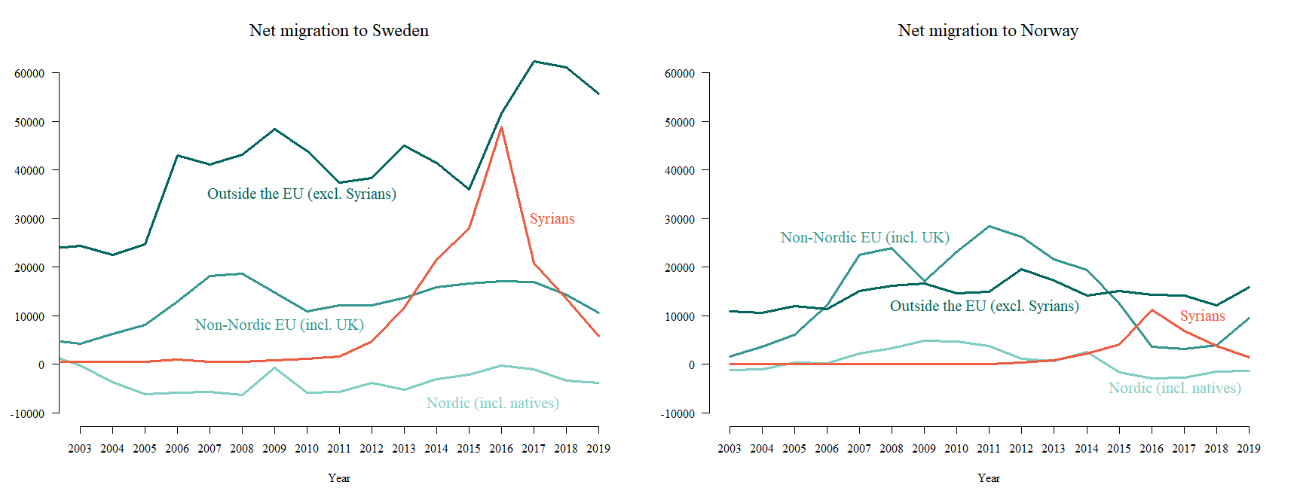


**Fig S2B: Net migration of Syrians and other groups of citizens to Sweden (left) and Norway (right), 2003-2019.**

Sources: Statistics Sweden and Statistics Norway.
