# Supplementary figures and images for: How Assad changed population growth in Sweden and Norway: Syrian refugees’ impact on Nordic national and municipal demography
Source: PLoS One. 2021 Jan 20;16(1):e0244670. doi: 10.1371/journal.pone.0244670 (PMC7816981; doi:10.1371/journal.pone.0244670)

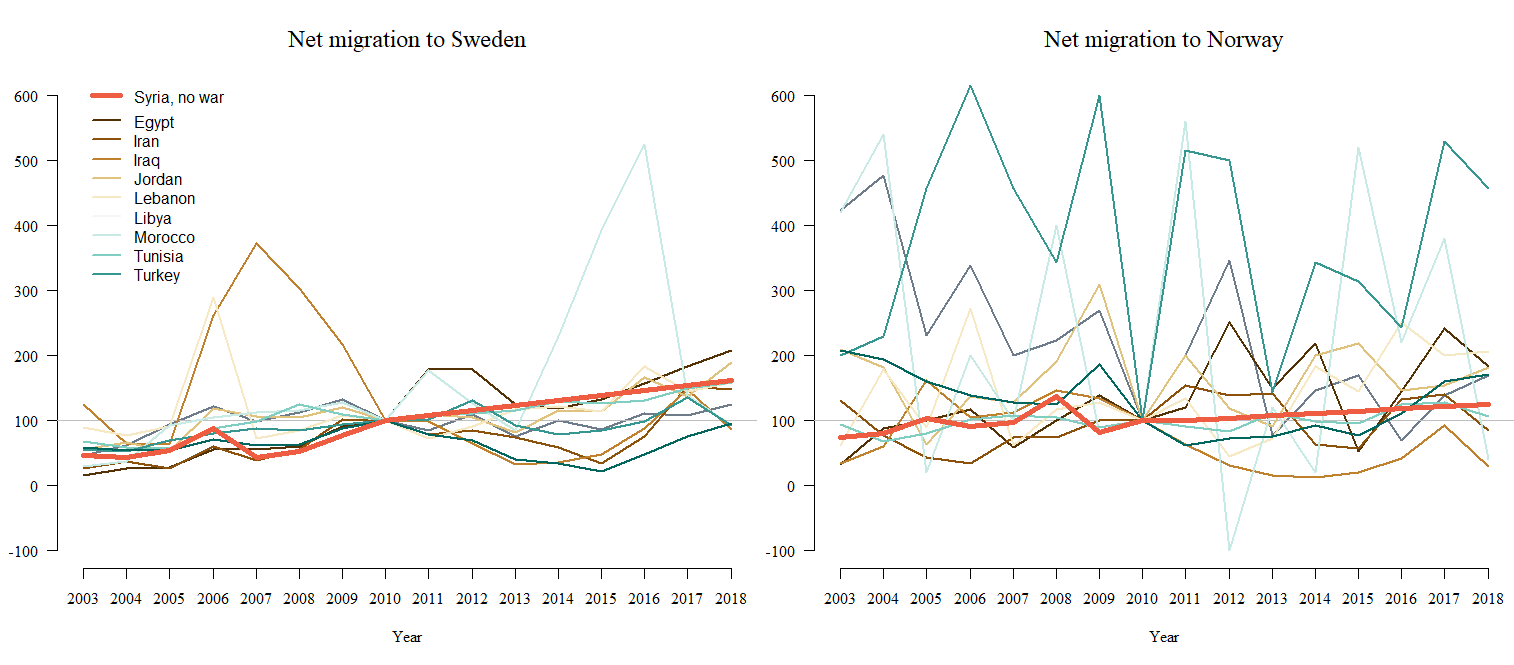

Supplement: S1 Fig — (TIF) [file pone.0244670.s005.tif]

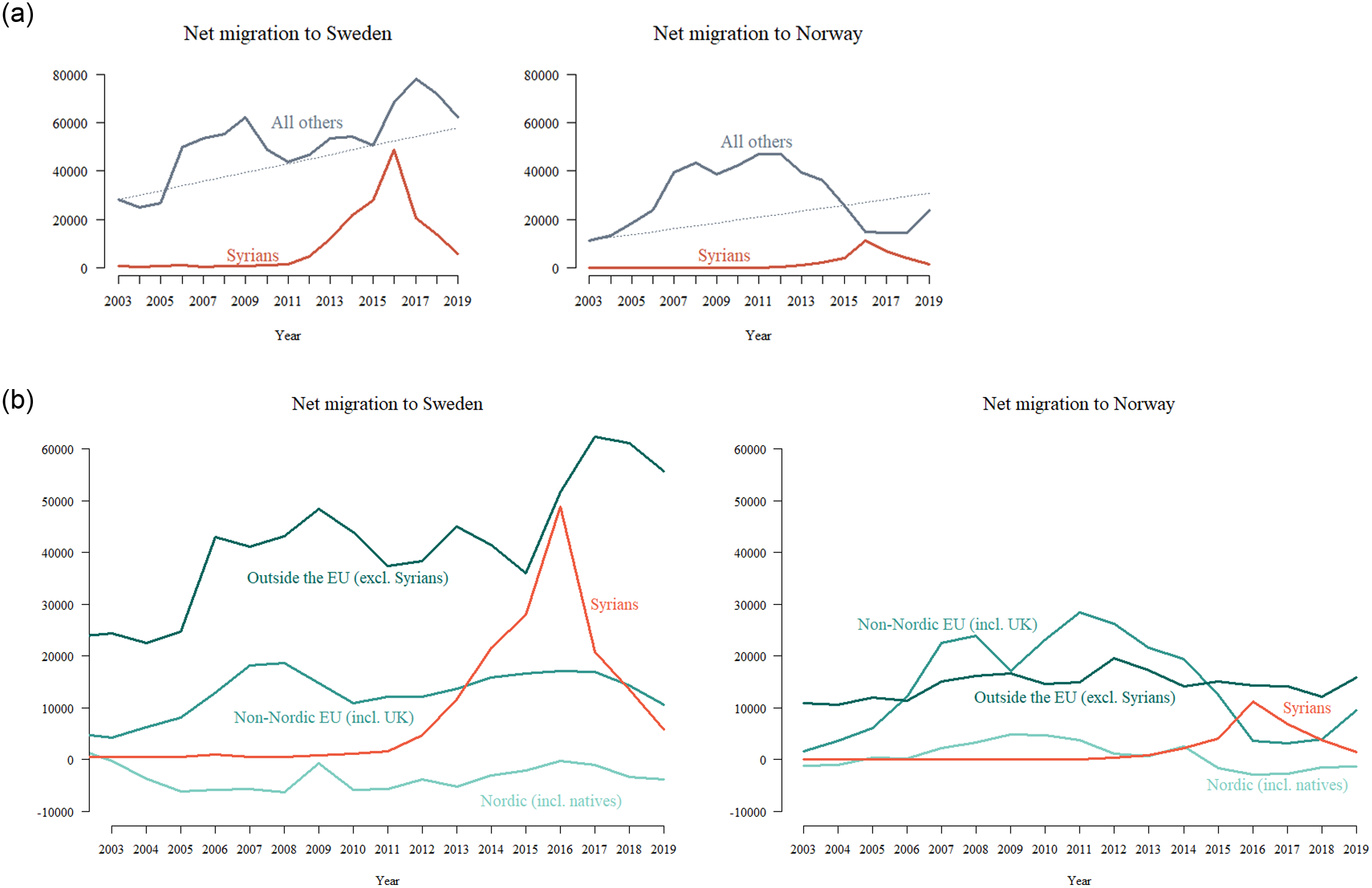

Supplement: S2 Fig — (TIF) [file pone.0244670.s006.tif]
